# A Hybrid Approach for Turning Intention Prediction Based on Time Series Forecasting and Deep Learning

**DOI:** 10.3390/s20174887

**Published:** 2020-08-28

**Authors:** Hailun Zhang, Rui Fu

**Affiliations:** 1 School of Automobile, Chang’an University, Xi’an 710064, China; Iszhanghailun@outlook.com; 2 Key Lab of Vehicle Transportation Safety Technology, Ministry of Transport, Chang’an University, Xi’an 710064, China

**Keywords:** advanced driver assistance system, autonomous vehicle, driving intention prediction, online time series prediction, bidirectional long short-term memory network

## Abstract

At an intersection with complex traffic flow, the early detection of the intention of drivers in surrounding vehicles can enable advanced driver assistance systems (ADAS) to warn the driver in advance or prompt its subsystems to assess the risk and intervene early. Although different drivers show various driving characteristics, the kinematic parameters of human-driven vehicles can be used as a predictor for predicting the driver’s intention within a short time. In this paper, we propose a new hybrid approach for vehicle behavior recognition at intersections based on time series prediction and deep learning networks. First, the lateral position, longitudinal position, speed, and acceleration of the vehicle are predicted using the online autoregressive integrated moving average (ARIMA) algorithm. Next, a variant of the long short-term memory network, called the bidirectional long short-term memory (Bi-LSTM) network, is used to detect the vehicle’s turning behavior using the predicted parameters, as well as the derived parameters, i.e., the lateral velocity, lateral acceleration, and heading angle. The validity of the proposed method is verified at real intersections using the public driving data of the next generation simulation (NGSIM) project. The results of the turning behavior detection show that the proposed hybrid approach exhibits significant improvement over a conventional algorithm; the average recognition rates are 94.2% and 93.5% at 2 s and 1 s, respectively, before initiating the turning maneuver.

## 1. Introduction

**Figure 1:**
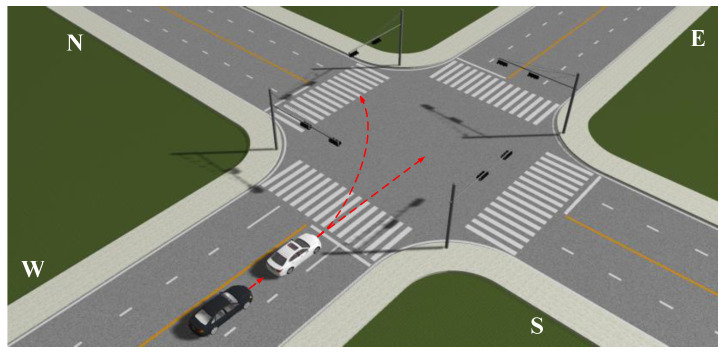
The scene of a vehicle entering the intersection.

With the widespread implementation of advanced driver assistance systems (ADAS) and the rapid development of artificial intelligence, autonomous driving has become a reality [1,2,3,4]. This development means that in the future, a mixed environment will be inevitable. Human-driven vehicles, autonomous vehicles, as well as connected vehicles, will travel together on roads. Many scholars have begun to study the characteristics of safe driving of mixed traffic and its impact on drivers [5,6,7,8,9,10,11]. It is well-known that intersections represent bottlenecks in urban traffic, reducing traffic efficiency. Due to the complex characteristics of intersections, the accident rate at or near this location is relatively high [12,13]. Numerous collisions and fatal accidents occurred at intersections in the United States, where an estimated 45% of injury crashes and 22% of roadway fatalities are intersection-related [14]. According to the EU community road accident database, in the past decade (2001–2010), intersection-related fatalities accounted for more than 20% in the EU [15]. The inability of drivers to assess correctly and/or observe dangerous situations is believed to be a major factor in these accidents [16]. For example, in Figure 1, the traffic light is green, allowing vehicles on East Street and West Street to pass. The white vehicle on West Street will turn left, followed by a black autonomous vehicle behind it, ready to drive straight through the intersection. The black vehicle needs to infer the turning behavior of the white vehicle to plan a safe driving path and minimize the acceleration or deceleration rate to improve passenger comfort and reduce emissions [17]. Therefore, the accurate and early detection of the driving intentions of surrounding vehicles is crucial to prevent traffic accidents and improve ride comfort for new-generation ADAS systems. The earlier the detection occurs, the earlier the system can intervene, and every millisecond is critical for safe driving.

In this paper, we propose a method for predicting and recognizing vehicle turning behavior at intersections using a combination of time series prediction and deep learning networks, which can predict the intention of the vehicle before the turning maneuver is initiated. In recent years, scholars conducted extensive research on driver behavior recognition or intention prediction [18,19,20,21,22,23]. Driving behavior is considered a continuous time-varying dynamic process [24,25], which is consistent with our general cognition. The problem of driving behavior recognition/inference/estimation is transformed into a problem of classification, recognition, or prediction of a time series. Many studies have used machine learning algorithms to analyze driving behavior, such as a continuous hidden Markov model (CHMM), Gaussian mixture model (GMM) [24,25,26,27,28,29], support vector machine (SVM) [30,31], back-propagation (BP) neural network [18,32], random forest, and Adaboost algorithm [33]. Specifically, in [24,27], an algorithm combining an HMM and Bayesian filtering (BF) was proposed to model the vehicle behavior while entering the intersection and performing a lane change. In [26], the author used the steering wheel angle and the steering wheel angle velocity as inputs for model training to develop the CHMM model. In [28], a CHMM and GMM were combined to model lane change and lane-keeping behaviors, respectively. The authors used public data and highway driving as a scenario to recognize and predict the lane change behavior of the target vehicle from the perspective of the host vehicle. In [25,29], a hybrid-state system (HSS) and HMM framework were integrated to model vehicle turning behavior at an intersection. In the HSS, the driver’s decision was modeled as a discrete state system, and the vehicle dynamics were modeled as a continuous state system. The SVM is a popular algorithm for classification problems, but it cannot model a time series. In [30,31], the objective was to classify driving behavior. The author concatenated different meta features of different times in the window to create a feature vector of fixed length or used the means and variances of the data collected in the sliding time window to replace individual measurements. In reference [32], the author developed a BP neural network prediction model of driver lane change behavior using vehicle movement data, relative motion data of the vehicle of interest and surrounding vehicles, and head movement data of the drivers. Another study [33] used ensemble learning methods to model lane changing driving behavior for the first time. The results showed that both methods provided high classification accuracies and low false alarm rates.

In existing research on driving behavior modeling, the HMM+GMM algorithm is most commonly used because the HMM can model time series of any length and infer unobserved (hidden) states. The GMM can model continuous observations using multiple probability density functions. As a result of the recent success of deep learning in image classification, speech recognition, and other fields, many researchers have begun to use this method for driving behavior recognition [34,35,36]. A long short-term memory (LSTM) model was proposed in [37] to increase the long-term dependency property and overcome the problem of gradient descent. LSTM is a variant of RNN that can capture and model long-term dependence in time series data. In [38,39,40], LSTM models were developed to infer the driving intention of vehicles approaching intersections. Since an LSTM can be trained in a sequence-to-sequence prediction manner, it can predict the trend of a period of time in the time series, enabling to predict the future activities of drivers [41].

The following conclusions can be drawn from the reviewed literature. First, regarding lane-changing behavior, what happens from the intent to the execution of the behavior is the result of the interaction between the driver and the surrounding environment. However, changes in the environment, such as pedestrians, front vehicle cut-ins, and motorcycle interference, are often less likely to modify the driver’s turning intention. Generally, the behavior of surrounding vehicles will only affect the steering maneuver, and it is difficult to reverse the driver’s turning intentions [42]. Intention prediction refers to recognizing the driving maneuver before the initiation of the actual maneuver, and behavior recognition refers to recognizing the driving maneuver in the early stage after the initiation of the maneuver. Since the behavior of the vehicle at the intersection is related to the driver’s task level, it is difficult to infer indirectly the driver’s intention through changes in the environment, such as the lane change behavior recognition that was proposed in [28]. When the driver’s destination is unknown, it is difficult to predict the turn behavior, similar to navigation software. However, different drivers have different driving preferences and characteristics, and the effects of their actions on the kinematics of the vehicle often reflect the driver’s intentions [15,43]. We can estimate the driver’s future activity from motion information and trajectory information of the vehicle approaching the intersection for a certain period of time, or recognize the activity in time when the vehicle exhibits early turning behavior characteristics. Second, empirical analysis has shown that if ADAS or autonomous vehicles cannot recognize the driving behavior of surrounding vehicles, the system cannot plan a safe and comfortable route in real-time. Most of the above studies were conducted from the perspective of the host vehicle, and the results may not be applicable to the behavior prediction and recognition of surrounding vehicles because the eye movement and facial tracking data of the drivers of the surrounding vehicles cannot be obtained [44,45]. In addition, most research on driving behavior recognition at intersections recognizes turning behaviors in the early stages after the maneuver has started [24,25,29,31]. Whereas references [15,38,39], and [40] claim to achieve intent prediction, ADAS or autonomous vehicles cannot record extensive historical data of the target vehicle to achieve intent prediction. During actual driving, the prediction of the future situation of the surrounding vehicles is often more in line with the driver’s preference, and the intention of the surrounding vehicles is inferred based on this [3]. However, in the literature, the prediction of car trajectories has mostly used offline algorithms, including the recurrent neural network (RNN), which is a deep learning network, LSTM [36,46], the interacting multiple model (IMM) [3], and the potential field method [47]. None of these methods meet the real-time requirements of the system, and the intuitive observation of the speed often has a significant effect on predicting the intention of the vehicle [42,48]. It is preferred to obtain the speed of the surrounding vehicles through on-board sensors. Furthermore, most studies that recognized or predicted driving behavior were essentially predicting time series or vector data based on variables that characterize the driving behavior, such as trajectory, speed, and acceleration. It should be noted that the vector data in previous studies is obtained using average or variance processing on the multi-dimensional variables in the time window.

**Figure 2:**
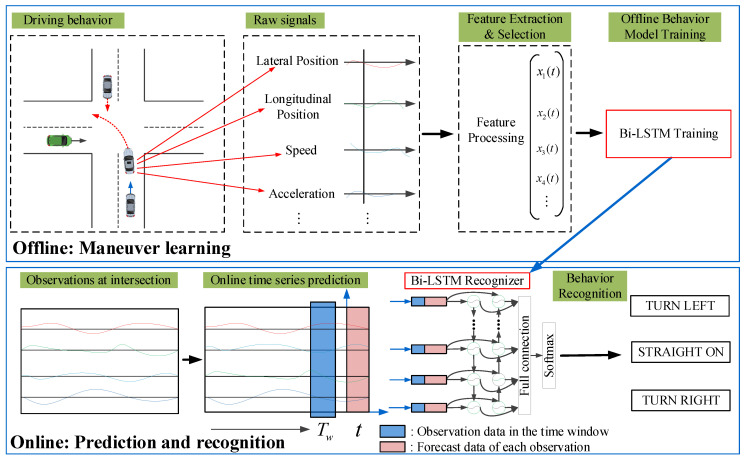
Schematic of intersection behavior prediction and recognition.

As stated, the main problem to be solved in this article is the early prediction of the driver’s intention before the actual turning maneuver begins. We regard the trajectory and kinematic characteristics of the vehicles as time series and consider the temporal context of these parameters. Based on the literature review and the concept of classification and prediction of time series [49,50], we propose a method for turning behavior prediction and recognition using online time series forecasting and deep learning classification. The proposed driver behavior prediction system comprises two layers, including offline behavior learning via a high-level behavior model and online behavior prediction via low-level vehicle state prediction. The schematic is shown in Figure 2. First, the driving behaviors at the intersection are learnt using a deep learning network. A bidirectional long short-term memory (Bi-LSTM) model is developed to recognize the turning behavior using time series data of the motion parameters and derived parameters characterizing vehicle behavior and intention. Second, an online autoregressive integrated moving average (ARIMA) time series prediction algorithm is used to predict the variables that characterizes the turning behavior. Then, the predicted data are used by the Bi-LSTM to predict drivers’ turning behavior.

To the best of the authors’ knowledge, this work is the first attempt to integrate online time series forecasting and behavior recognition for driving behavior prediction. The proposed framework is validated using the open-source next-generation simulation (NGSIM) dataset, which is based on real-world driving experiments that were conducted by the Federal Highway Administration’s (FHWA) and provided promising performance.

The main contributions of this study are as follows.

- The purpose of this research is to realize the intention prediction of the vehicle before the start of the steering maneuver, and propose a framework that combines time series prediction and deep learning methods to apply to the new generation of ADAS or future autonomous vehicles. The framework uses online prediction algorithms to reflect the driving intention of the vehicle in the prediction window, and achieves high recognition accuracy and modeling of vehicle kinematics that indicate steering behavior through Bi-LSTM.
- Since the variables representing the driving behavior are time series data, a novel vehicle behavior prediction method is proposed that combines the ARIMA with an online gradient descent (OGD) optimizer. This method allows for predicting the driving intention without reducing the recognition rate.

The remainder of this paper is organized as follows. Section 2 details the proposed online ARIMA and Bi-LSTM hybrid approach. The experimental data and the data processing and analysis methods are described in Section 3. The experimental results are described in Section 4. Finally, the conclusions are provided in Section 5, and potential future studies are outlined.

## 2. Framework for Turning Behavior Recognition

Survey results have shown that the dominant factor causing traffic accidents is human error. More than 90% of traffic accidents are partially caused by human error, and 57% of accidents are entirely caused by human error [48]. Perception of danger has always been a focus of ADAS development. The prediction of the driving intentions based on the movement of surrounding vehicles in real-time has been a challenge for ADAS systems.

In this section, we describe the methods used in the proposed framework. The system is divided into two parts. In the offline learning phase, a Bi-LSTM model is used to learn the extracted turning behavior. The online phase takes place in two steps. Multiple time series variables that characterize the turning behavior are predicted using the online ARIMA algorithm, and the predicted data and the derived parameters are used in conjunction with the offline training data of the turning behavior to recognize the turning behavior.

### 2.1. Bi-LSTM

We know from the literature that when vehicles approach an intersection, the historical information hidden in the vehicle’s motion parameters often reflects the driver’s turning intention, and previous information often has a considerable impact on the recognition result [15,40,43]. Therefore, it is preferred to use a model that considers the behavioral characteristics of the vehicle for an extended period before approaching the intersection to detect the turning behavior.

**Figure 3:**
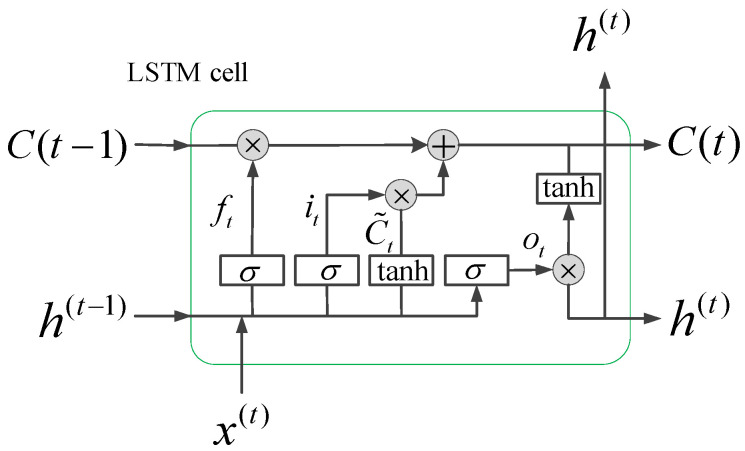
Internal structure of a long short-term memory (LSTM) cell.

RNNs use historical information to assist in current decision-making. However, earlier signals have less important information than more recent signals and RNNs cannot solve the long-term dependence problems. An LSTM network with its unique gate structure not only solves the problem of long-term dependence but also addresses gradient vanishing or explosion that occur when RNNs are used to process time series and can be used to model arbitrary time series [41]. Each gate has its own weight matrix and time lag. A single LSTM unit is shown in Figure 3.

**Figure 4:**
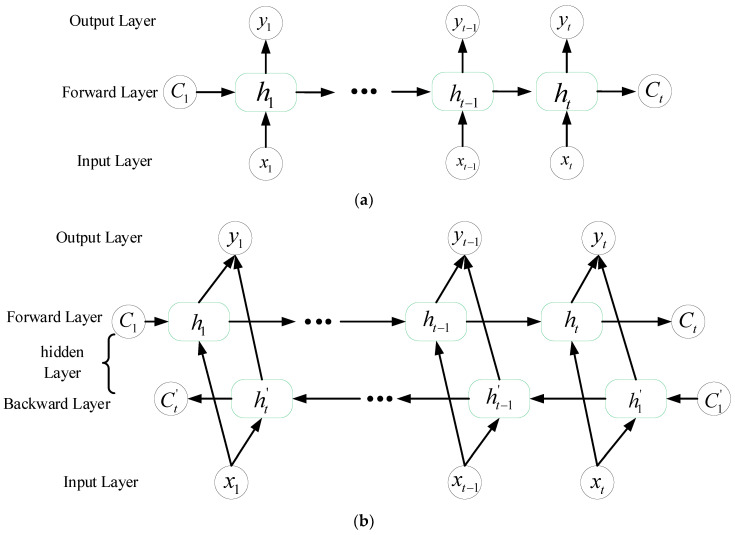
Illustration of a LSTM model (**a**) and a bidirectional (Bi-LSTM) model (**b**).

A graphic illustration of a standard LSTM network is depicted in Figure 4a. The *i* is an input gate that controls the size of the new content added to the memory. The *f* is a forget gate that determines the amount of memory that should to be forgotten. The *o* is an output gate that adjusts the amount of output memory content. The *c* is the cell activation vector, which consist of the partially forgotten previous memory *c_t_*_−1_ and modulated the new memory ${\tilde{c}}_{t}$. t represents the t-th moment [51].

(1) $$\begin{array}{c} i_{t}=\sigma \left(W_{xi}x_{t}+W_{hi}h_{t-1}+b_{i}\right)\\ f_{t}=\sigma \left(W_{xf}x_{t}+W_{hf}h_{t-1}+b_{f}\right)\\ o_{t}=\sigma \left(W_{xo}x_{t}+W_{ho}h_{t-1}+b_{o}\right)\\ {\tilde{c}}_{t}=tanh\left(W_{xc}x_{t}+W_{hc}h_{t-1}+b_{c}\right)\\ c_{t}=f_{x}⊗c_{t-1}+i_{t}⊗{\tilde{c}}_{t}\\ h_{t}=o_{t}⊗\tan h(c_{t}) \end{array}$$

where $i_{t}$, $f_{t}$, $o_{t}$, and $c_{t}$ represent the values of *i*, *f*, *o* and *c* at time *t*, respectively. *y_t_* is the output of the output layer at time *t*. *x_t_* is the current input vector. *h_t_* is the hidden layer vector at time *t*, which contains all the outputs of the LSTM. The *W* represents the self-updating weight of the hidden layer, and *b* represents the bias vector. The σ is the sigmoid activation function, and tanh is the hyperbolic tangent function. The operator ⊗ is the dot multiplication operation. All gate values and hidden layer outputs are in the range (0, 1).

In [27], lane change intention recognition is considered a speech recognition problem. We know that when filling vacancies in a sentence, it is necessary to grasp the words before the vacancy; however, the words after the vacancy must also be considered to predict an outcome that is in line with the situation and accurate. The Bi-LSTM solves these types of problems. In the example of the sequence, the network connects the same output from two LSTM networks that is learned and modeled from both ends of the sequence. The forward LSTM obtains past data information of the input sequence, and the backward LSTM obtains future data information of the input sequence [52]. Therefore, the network processes information from both ends of the time series and not only considers the influence of historical data but also that of future states, thereby significantly increasing the generalization ability of the model. The model structure of the LSTM is shown in Figure 4b. The formula is expressed as follows:

(2) $$\begin{array}{c} \vec{h_{t}}=\vec{LSTM}\left(h_{t-1},x_{t},c_{t-1}\right),t\in \left[1,T\right]\\ \overset{\leftarrow }{h_{t}}=\overset{\leftarrow }{LSTM}\left(h_{t+1},x_{t},c_{t+1}\right),t\in \left[T,1\right]\\ H_{t}=\left[\vec{h_{t}},\overset{\leftarrow }{h_{t}}\right] \end{array}$$

where *H_t_* is the hidden state of the Bi-LSTM at time *t*, including two the LSTMs that operate in the opposite directions $\vec{h_{t}}$ and $\overset{\leftarrow }{h_{t}}$.

### 2.2. Online ARIMA

As mentioned before, the vehicle’s driving trajectory and motion feature parameters can be considered a dynamic process in a time series. On this basis, the ARIMA algorithm is adopted. Because ARIMA has good statistical properties and excellent flexibility, it is one of the most commonly used linear models for time series prediction [53].

The proposed online prediction model processes the collected vehicle trajectory and motion feature parameters in the order in which they arrive and simultaneously updates the model parameters. Subsequently, the trajectory and motion feature parameters are predicted for a period based on the constantly updated model. This type of processing is consistent with the characteristics of quantitative observation sequences over time.

Since they are influenced by the driver, the trajectory and motion feature parameters of the vehicle are generally not in a steady-state and may contain some deterministic trends. The differential method is an effective method for dealing with high sequential correlation. The ARIMA model is defined as follows:

(3) $$D^{d}X_{t}=\overset{q}{\underset{i=1}{\sum }}\beta_{i}\epsilon_{t-i}+\overset{k}{\underset{i=1}{\sum }}\alpha_{i}D^{d}X_{t-i}+\epsilon_{t}$$

where $D^{d}$ represents the *d*-th order difference, *D* is expressed as a differential symbol, $X_{t}$ denotes the observation at time *t*. $\sum_{i=1}^{q}\beta_{i}\epsilon_{t-i}$ represents the moving average (MA) model. $\sum_{i=1}^{k}\alpha_{i}D^{d}X_{t-i}$ is the autoregressive (AR) model. $\epsilon_{t}$ is the zero-mean noise term. α and β are weight vectors. *k*, *d*, and *q* are parameterized terms.

We assume that $X_{t}$ satisfies the ARIMA (*k*, *d*, *q*) model, the prediction of $X_{t}$ over time can be achieved by an inverse differential process. The predicted value at time *t* + 1 as ${\tilde{X}}_{t}$ can be expressed by the following equation:

(4) $${\tilde{X}}_{t}=D^{d}{\tilde{X}}_{t}+\overset{d-1}{\underset{i=0}{\sum }}D^{i}X_{t-1}$$

For the ARIMA model of Equation (3), due to the existence of a noise term $\epsilon_{t}$, the existing online convex optimization technique cannot be applied to estimate the coefficient vector. Therefore, an ARIMA (*k* + *m*, *d*, 0) model that approximates the original ARIMA (*k*, *d*, *q*) and has no noise terms is designed. The expression is as follows,

(5) $${\tilde{X}}_{t}\left(\gamma^{t}\right)=\overset{k+m}{\underset{i=1}{\sum }}\gamma_{i}D^{d}X_{t-i}+\overset{d-1}{\underset{i=0}{\sum }}D^{i}X_{t-1}$$

where *m* is a constant, and γ is the new weight vector.

The loss function is:

(6) $$\ell_{t}^{m}\left(\gamma^{t}\right)=\ell_{t}\left(X_{t},{\tilde{X}}_{t}\left(\gamma^{t}\right)\right)=\ell_{t}\left(X_{t},\left(\overset{k+m}{\underset{i=1}{\sum }}\gamma_{i}D^{d}X_{t-i}+\overset{d-1}{\underset{i=0}{\sum }}D^{i}X_{t-1}\right)\right)$$

The goal of online ARIMA learning is to minimize the sum of losses over multiple iterations. After eliminating the noise term, we use the OGD method as an online convex optimization solver in this study [54]. More details on the OGD optimization technique can be found in [55].

The prediction algorithm needs a large dataset to update the model and cope with the rapidly changing vehicle trajectory and motion parameters to predict the future trend accurately. As the acquired data volume increases, the prediction result approaches the actual value.

## 3. Experimental Data

The mathematical model discussed in Section 2 requires a large amount of data for successful model training. The data must also be carefully selected to enable researchers to train models that correspond to the expected vehicle events.

### 3.1. Data Description

We use the open-source NGSIM dataset to verify the performance of the proposed hybrid method; the dataset was provided by the FHWA NGSIM project. This dataset has been widely used to develop and test various models [28,33,40,56,57,58]. A literature review indicated that most scholars investigated lane changes using the US-101 and I-80 dataset, but few had analyzed the turning behavior at intersections using this dataset. The NGSIM Lankershim and Peachtree street datasets are used in this work to develop the behavior recognition model for vehicles entering intersections. The behavior of the vehicle includes turning left (TL), turning right (TR), and going straight (GS). 

**Figure 5:**
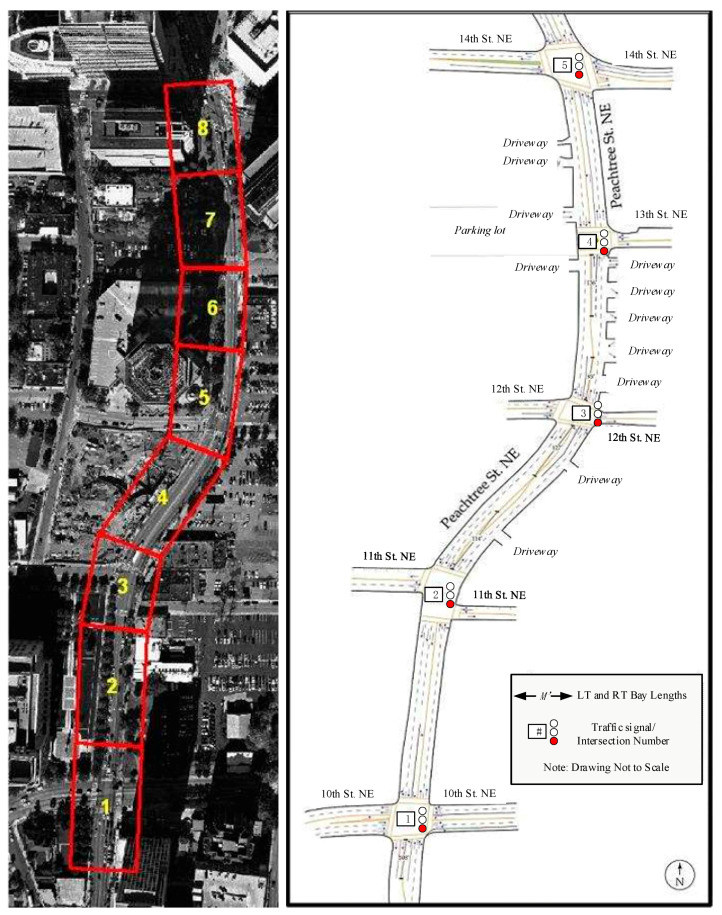

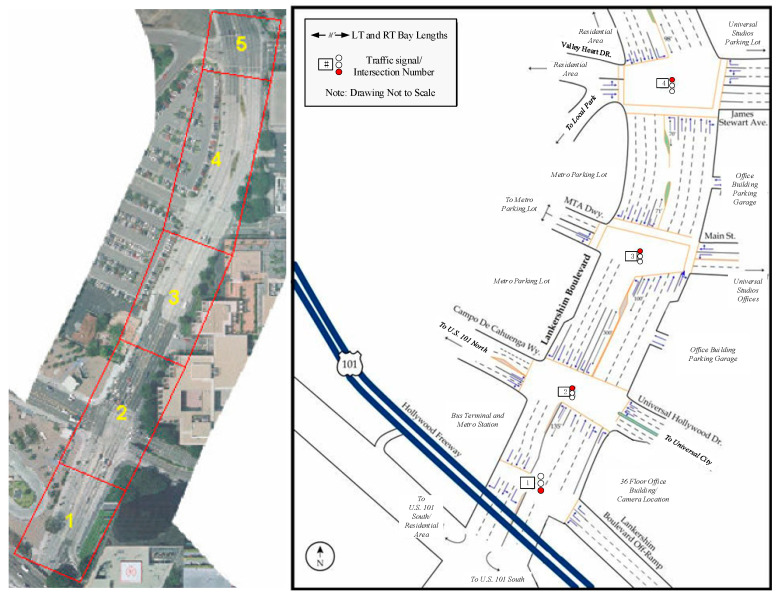
(**a**) Lankershim Boulevard. (**b**) Peachtree Street.

Figure 5 is a schematic diagram of the intersection area investigated in this work. The red boxes and labels in the left picture of Figure 5a,b are the study area and camera coverage, and the right picture is the schematic diagram of the study area. Figure 5a shows Lankershim Boulevard, an artery running primarily north-south in Los Angeles, California. The speed limit on Lankershim Boulevard is 35 mph. Figure 5b shows Peachtree Street, which is a main road in Atlanta, Georgia; it runs from north to south, with a speed limit of 35 mph. As shown, there are four intersections on Lankershim and five intersections on Peachtree. Lankershim road is wide and is approximately 2100 feet in length, and Peachtree Street is narrow and is approximately 1600 feet in length. It also includes T-shaped intersections. The Lankershim data were collected on 16 June 2005 from 8:45 to 9:00 in the morning during the peak commuting period. The collection time of the Peachtree street data was from 12:45 to 1:00 and 4:00 to 4:15 on the afternoon of 8 November 2006. 

The dataset contains information on the vehicle’s lateral and longitudinal position, speed, acceleration, vehicle type, lane ID, and time/space headway, which were obtained from the video trajectory data at a resolution of 10 frames/s using a tracking algorithm [33]. In this work, we used some of the available variables.

### 3.2. Data Extraction

**Figure 6:**
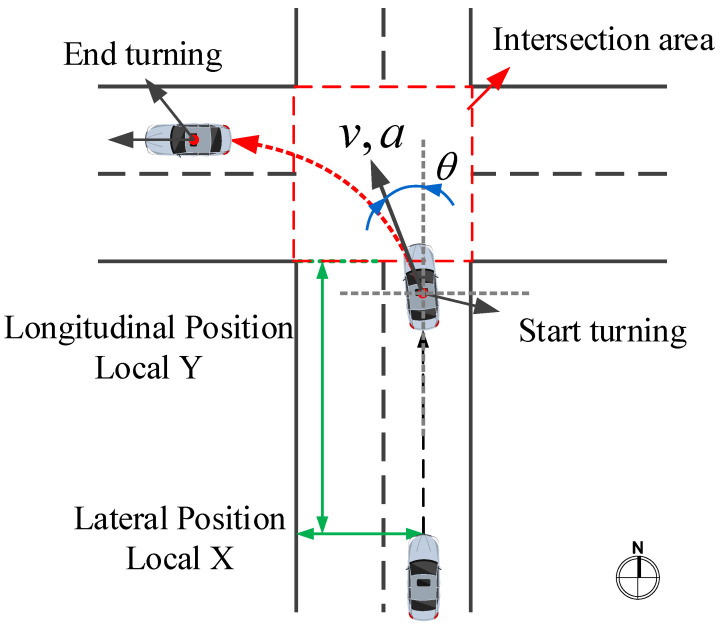
The process of a vehicle entering the intersection.

The purpose of this work is to recognize the TL, TR, or GS behavior of the vehicle when approaching the intersection before initiating the maneuver. In other words, we predict the future maneuver of the vehicle rather than classify the ongoing turning events to provide support for new generation ADAS or future intelligent vehicles or intelligent transportation systems. The process of the vehicle entering the intersection and turning includes the following sub-processes: first, the vehicle starts to decelerate, then the driver turns the steering wheel to change the vehicle’s direction of travel, and when the direction meets the driver’s needs, the driver starts to accelerate and drives away from the intersection. Figure 6 shows the process of a vehicle traveling from south to north, entering an intersection and turning left.

**Figure 7:**
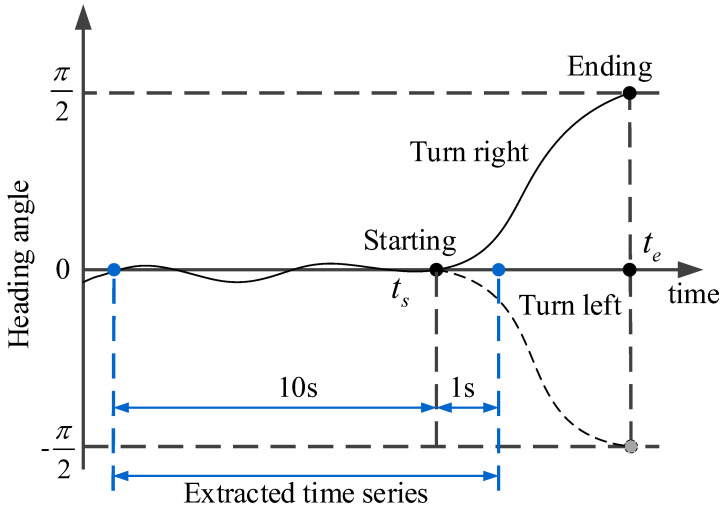
The process of entering the intersection.

Due to the influence of the length of the intersection or the habits of the driver, the process of passing through the intersection has different durations. The turning process needs to be extracted to ensure that the differences between the samples are not excessive and to shorten the training time of the model. In this work, the extracted sample time for the vehicle approaching the intersection is 11 s. The “Movement” feature in the dataset is used to indicate the vehicle’s current maneuvering state. When the value of movement is 1, it denotes GS, 2 denotes TL, and 3 denotes TR. However, when the trajectory information of the vehicle is combined with these data, it is found that the vehicle state indicated by this value is not accurate, and it is difficult to determine the starting time and ending time of the vehicle turning maneuver accurately. The heading angle is often regarded as a crucial parameter of the vehicle during the turning maneuver. The starting and ending time of a turning maneuver can be determined by observing the course of the heading angle. Normally, after a turning maneuver is completed, the vehicle heading angle has changed by about 90°. According to vehicle kinematics, the heading angle θ defined in this work is calculated as follows:

(7) $$\theta =\arctan\left(\frac{\dot{y}}{\dot{x}}\right)=\arctan\left(\frac{v_{y}}{v_{x}}\right)$$

where *x* and *y* represent the lateral and longitudinal coordinates of the vehicle, and *v_x_* and *v_y_* are the lateral and longitudinal speeds of the vehicle, respectively. The starting time *t_s_* is the initial time of the increasing or decreasing part on the curve, and the ending time *t_e_* is the end time of the increasing or decreasing part on the curve [24], as shown in Figure 7. It should be noted that we define the heading angle as a negative value when TL and a positive value when TR (Figure 7), and the heading angles corresponding to the starting time and ending time set here are not exactly 0° and 90° or −90° because there are fluctuations. The extraction process follows the following criteria:

1. Identify the ID of the vehicle TL or TR;
2. Calculate the heading angle of the vehicle based on the trajectory information of the vehicle;
3. Search the starting time *t_s_* when the vehicle begins to turn and mark it;
4. Using the *t_s_* as a reference, 11 s is extracted from the time series of the entire turning process, including the time series of 10 s before *t_s_* and 1 s after *t_s_*.

After this extraction process and manual selection, invalid data are eliminated, such as short-duration turning or GS maneuver samples; eventually, 2993 sets of sample data are extracted. The data of the different road sections and different time periods are summarized in Table 1. The number of maneuvers in the Peachtree dataset is relatively small because we eliminated many invalid data and short-duration data.

### 3.3. Input and Output Variable

**Figure 8:**
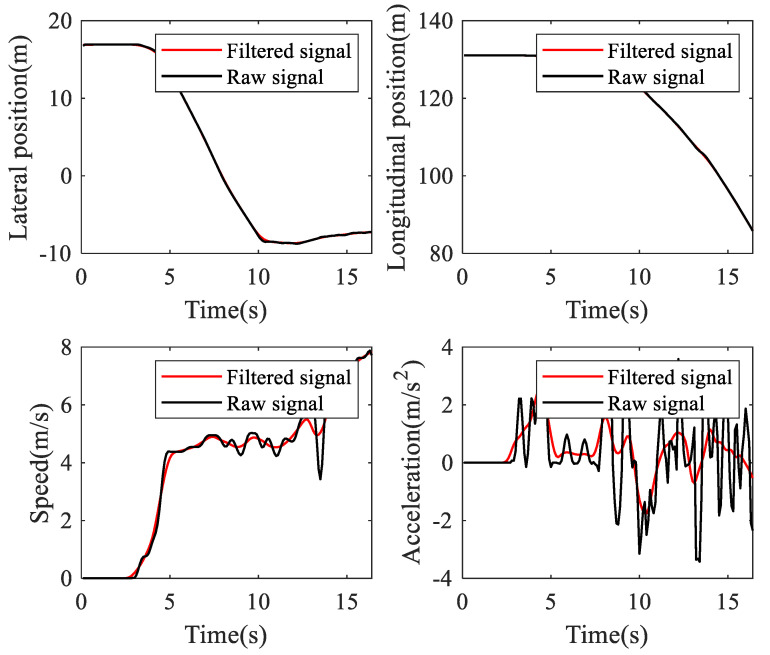
The process of entering the intersection.

The raw dataset of Lankershim and Peachtree cannot be used directly in the model because it has noise and errors. The locally weighted scatterplot smoother (LOWESS) algorithm was adopted to smooth the extracted data [59], as shown in Figure 8. The speed, acceleration, and the lateral and longitudinal position of the TL maneuvers are filtered. The use of filtered data does not only accelerate the convergence of the loss function during the model training phase but also makes the online prediction algorithm more stable. Note that the distance and speed units in the original dataset are feet and feet per second, which are converted into international units of meters and meters per second.

The parameters used in this work to characterize the vehicle’s motion are the vehicle’s lateral and longitudinal positions, speed, and acceleration. As shown in Figure 6, Local X (*x*) indicates the lateral coordinate of the front center of the vehicle with respect to the left-most edge of the section in the direction of travel, Local Y (*y*) denotes the longitudinal coordinate of the front center of the vehicle with respect to the edge of the section in the direction of travel, *v* and *a* are the speed and acceleration of the vehicle, respectively. It should be noted that because vehicles will enter the intersection from all directions, the trajectory information of different vehicles in the dataset will be quite different.

The input data $X_{t}$ of the prediction algorithm consists of all observations from a particular trajectory segment at time *t*, and $Y_{t}$ is the output of its prediction. $x_{t}$ contains the lateral ($x_{t}$) and longitudinal ($y_{t}$) positions, as well as the speed ($v_{t}$) and acceleration ($a_{t}$), where:

(8) $$\begin{array}{c} X_{t}=\left[x_{t-h+1},x_{t-h+2},x_{t-h+3},\ldots ,x_{t}\right]\\ x_{t}=\left[x_{t},y_{t},v_{t},a_{t}\right]\\ Y_{t}=\left[y_{t+1},y_{t+2},y_{t+3},\ldots ,y_{p}\right]\\ y_{t}=\left[x_{t},y_{t},v_{t},a_{t}\right] \end{array}$$

Here, $X_{t}$ is the given historical observation sequence and *h* is the sequence length; $Y_{t}$ is the predicted sequence, and *p* is the predicted length.

In addition, the vehicle’s lateral motion parameters reflect the vehicle’s trajectory and status. Based on this, we use the local coordinates related to the road to calculate the parameters of the vehicle’s lateral motion state, including the lateral speed *v_x_*, lateral acceleration *a_x_*, and the key variable, i.e., the heading angle *θ*. The input of the Bi-LSTM behavior recognition model is $X_{t}^{B}$, where:

(9) $$X_{t}^{B}=[Y_{t},x_{t}^{\mathrm{lat}}]$$

Here, $x_{t}^{\mathrm{lat}}=\left[v_{xt},a_{xt},\theta_{t}\right]$.

### 3.4. Data Analysis

**Figure 9:**
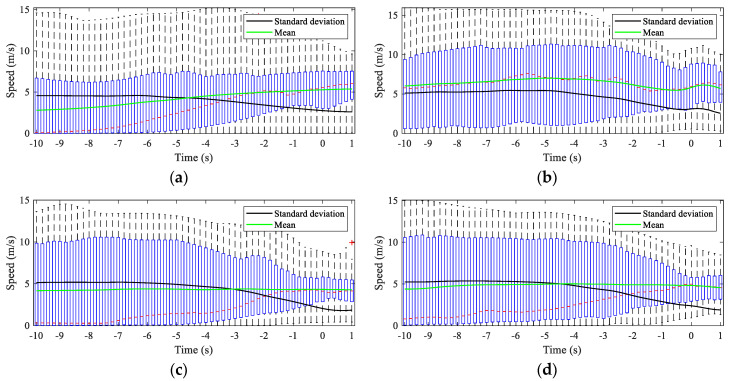
Boxplot of the speeds of the vehicle in the Lankershim and Peachtree datasets. (**a**) Statistics of the turn left (TL) speed in the Lankershim dataset. (**b**) Statistics of the turn right (TR) speed in the Lankershim dataset. (**c**) Statistics of the TL speed in the Pearchtree dataset. (**d**) Statistics of the TR speed in the Pearchtree dataset.

**Figure 10:**
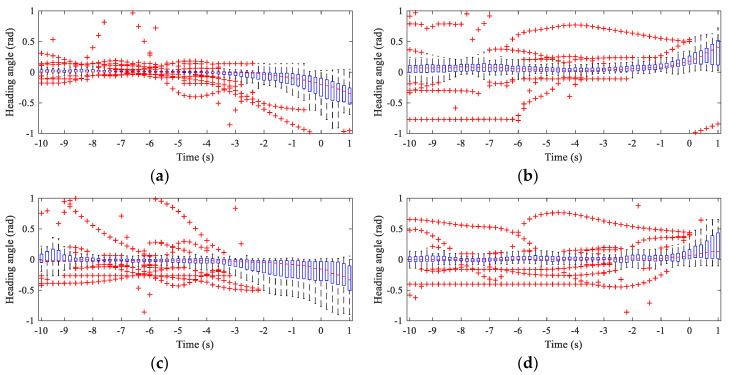
Boxplot of heading angles of the vehicles in Lankershim and Peachtree dataset. (**a**) Statistics of the TL heading angle in the Lankershim dataset. (**b**) Statistics of the TR heading angle in the Lankershim dataset. (**c**) Statistics of the TL heading angle in the Pearchtree dataset. (**d**) Statistics of the TR heading angle in the Pearchtree dataset.

Different drivers have different driving preferences or characteristics for different maneuvers at intersections [15]. The driving speed is often the critical parameter reflecting the driving characteristics of the driver. By observing the changing characteristics of the driving speed, it is often possible to detect driving intentions early. For turning maneuvers, the change in the heading angle, which is a crucial parameter, can often be used as a critical indicator to determine the start and end of the maneuver. The vehicle speed and heading angle of the samples extracted from different datasets are statistically analyzed to understand the vehicle’s turning maneuver, as shown in Figure 9 and Figure 10. The abscissa in the figures is time with an interval of 0.2 s, and 0 indicates the starting time of the turning maneuvers. Note that the speed and heading angle are statistically analyzed only from 10 s before *t_s_* to 1 s after *t_s_*, and each boxplot contains all sample subjects for each maneuver. The speed characteristics in Figure 9 show that the standard deviation starts to decrease at around −5 s, regardless of whether it is a left turn or a right turn, indicating that the driver may have started to execute his intention 5 s before the start of the turning maneuver, and that this was reflected by the change in speed. The mean values of the speed show that the driver on Peachtree is relatively stable when TL and TR, and the trend in the speed is similar. The speed of the vehicle on Lankershim before the start of the TL is significantly slower than that before the start of the TR, but when approaching the *t_s_*, the speeds of the two maneuvers are similar. The heading angle boxplots shown in Figure 10 indicate that the heading angle has a tendency to change from −3 s to −4 s before *t_s_*, although there are some outliers in the data. This result indicates that the driver started to control the vehicle’s heading 3 to 4 s before the initiation of the actual turning maneuver, such as merging or adjusting the vehicle’s position However, we can also see from the comparison of TL in Figure 10a,c, and TR in Figure 10b,d that different intersections have various impacts on the vehicle’s turning maneuver. Although the change in the heading angle is about 90°, this change differs for intersections of different lengths.

### 3.5. Training and Test Procedure

#### 3.5.1. Evaluation Index for the Online Prediction Algorithm

In this work, the mean absolute percentage error (MAPE) and the root mean square error (RMSE) are used as an indicator to reflect the forecasting accuracy of the proposed online prediction algorithm. The calculation formula of these two evaluation indexes are defined as follows:

(10) $$\begin{array}{c} MAPE=\frac{1}{n}\sum_{i=1}^{n}\frac{\left|y_{i}-{\hat{y}}_{i}\right|}{y_{i}}\times 100\%\\ RMSE=\sqrt{\frac{1}{n}}\sum_{i=1}^{n}{\left(y_{i}-{\hat{y}}_{i}\right)}^{2} \end{array}$$

where *n* is the total number of data, $y_{i}$ and ${\hat{y}}_{i}$ are the real value and predicted value at the *i*-th time, respectively.

#### 3.5.2. Training of the Behavior Recognition Model

The dataset was divided into training and testing datasets to develop and evaluate the model. In addition, five-fold cross-validation (CV) method is used to test the performance of the model. The extracted sample data are randomly and evenly divided into five folds; four folds are used to train the model, and one fold is used to evaluate the trained model. A total of five times are performed for such procedures. After implementing the test procedure, the model performances for detecting TL, TR, and GS are determined.

The receiver operating characteristic (ROC) curve was used to evaluate the model performance; this method evaluates the performance of a classifier by assessing the true positive rate (TPR) and false positive rate (FPR). The calculation formulas of the TPR and FPR are as follows:

(11) $$\begin{array}{c} TPR=\frac{TP}{TN+FN}\\ FPR=\frac{FP}{TN+FP} \end{array}$$

where *TP*, *TN*, *FP*, and *FN* are the true positives, true negatives, false positives, and false negatives, respectively. The function provided by Matlab is used to calculate the ROC curve of the model, and *TP*, *TN*, *FP*, and *FN* are calculated after obtaining the true class labels and predicted scores of the test samples. According to the ROC curve of the model, the recognition performance of the model can be compared for different methods or premises by calculating the value of the area under the curve (AUC), where the value range of AUC is from 0 to 1. The larger the AUC value, the better the model performance is.

In this work, the developed Bi-LSTM network consists of 4 layers of stacks, the number of hidden units in each layer is 128, the activation function of the fully connected layer is ReLU, and the dropout rate is 0.9. A BP algorithm with the Adam stochastic optimization method is used to train the network over time with a learning rate of 0.001. The networks are trained using batches of size 80, and the epoch is 100. Note that all networks and algorithms are implemented in MATLAB 2019a under the Windows 10 Operation System and are evaluated on a PC with the following configuration: Intel Core i7-8700CPU at 3.20 GHz with 16 GB of RAM.

## 4. Results and Discussion

In this section, the sample data extracted in Section 3 are used to verify the performance of the proposed hybrid method for predicting the turning behavior. As mentioned before, we first use an online prediction algorithm to predict the vehicle’s trajectory and state and use the predicted results as the input to the turning behavior recognition model to obtain the turning maneuver prediction results. The purpose is to predict the vehicle’s intention as early as possible before the initiation of the maneuver. The basic parameters extracted from the dataset that characterize the vehicle’s motion state include the lateral and longitudinal coordinates, as well as the speed and acceleration. Based on the above parameters, the derived parameters that intuitively characterize the vehicle’s lateral state are obtained; these include the lateral speed, lateral acceleration, and heading angle. Therefore, the online prediction algorithm only predicts the basic parameters of the vehicle, and the derived parameters can be calculated based on the prediction results, thereby reducing the workload of the prediction algorithm and shortening the calculation time.

### 4.1. Performance of the Online Prediction Algorithm

The use of an offline model to predict the vehicle’s future trajectory or motion state is often suitable for algorithm verification, but it does not meet the real-time requirements of the ADAS system, i.e., it cannot be used to evaluate the actual driving process. However, online prediction algorithms can achieve this, and they also meet people’s expectations for predicting the movement of surrounding autonomous vehicles in the future. This algorithm predicts the vehicle’s trajectory and movement in the future based on historical information, providing advanced prediction of the maneuver time. As mentioned in Section 2.2, in the algorithm, the model parameters are continuously updated based on the acquired data, and the future values are predicted based on the new model. In the beginning, the prediction algorithm has just received real-time data and has generated the model parameters. The data error based on the model prediction at this time will be relatively large; however, as more and more data enter the model, the prediction accuracy of the algorithm will increase. In this work, we do not start at the beginning of the extracted 11 s time series, but rather at 11 s before the *t_s_* until the end of the sequence, and we set the prediction horizon to 1.5 s. As a result, more accurate real-time data are available for the subsequent turning behavior recognition model. Note that entering an intersection from different directions will result in large differences in the trajectories, as mentioned previously. 

**Figure 11:**
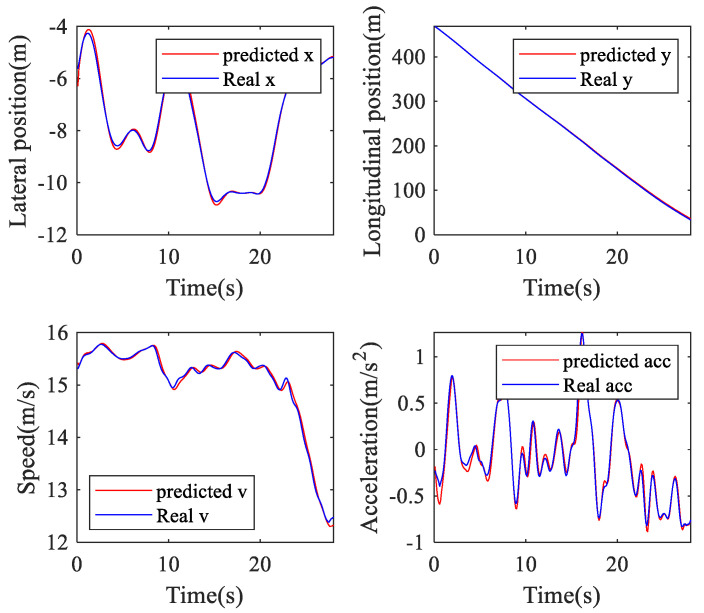
Going straight prediction.

**Figure 12:**
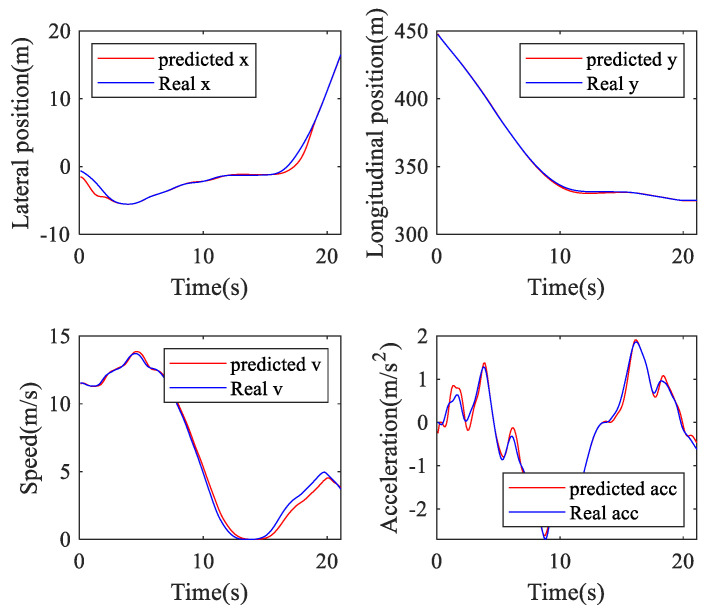
Turn left prediction.

**Figure 13:**
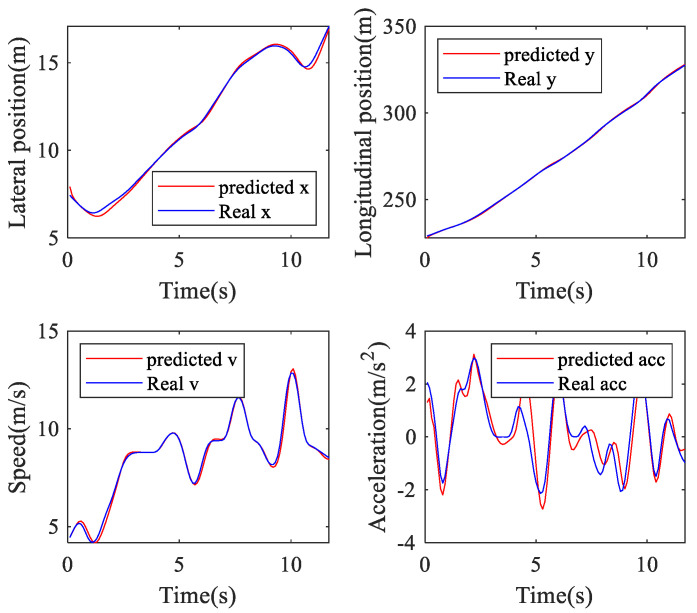
Turn right prediction.

In this work, the lateral position, longitudinal position, speed, and acceleration of the TL and TR processes are used to verify the performance of the online ARIMA prediction algorithm. Table 2 presents the RMSEs and MAPEs of these parameters. Figure 11, Figure 12 and Figure 13 are GS, TL, and TR processes randomly selected from the dataset, respectively. The performance of the proposed prediction algorithm by the RMSE in Table 2 and Figure 11, Figure 12 and Figure 13 illustrates that the prediction algorithm accurately predicts the driving trajectory and motion state of the vehicle. It can be seen from Table 2 and Figure 11, Figure 12 and Figure 13 that the algorithm can relatively accurately predict the trajectory and motion state of the vehicle in the predicted horizon, which shows that the algorithm can reflect the driving intention of the vehicle in the future to a certain extent.

### 4.2. Performance of the Hybrid Method for Turning Behavior Recognition

**Figure 14:**
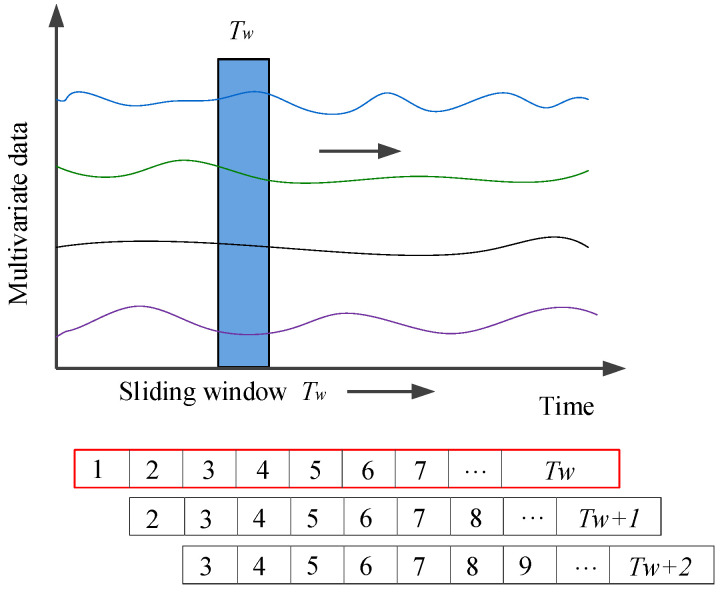
Sliding time window.

The performance of the proposed hybrid method is evaluated in terms of the recognition accuracy and behavior recognition time. A sliding time window is used to maximize the use of the data, as shown in Figure 14. The moving step is 0.1 s, i.e., each time the window moves forward, the data in the subsequent and previous windows always contains the same *T_w_* − 0.1 s information. Since the window is relatively wide, the real-time performance of the model is affected, and large computing memory is required. After a comparative test, *T_w_* is set to 1.5 s.

**Figure 15:**
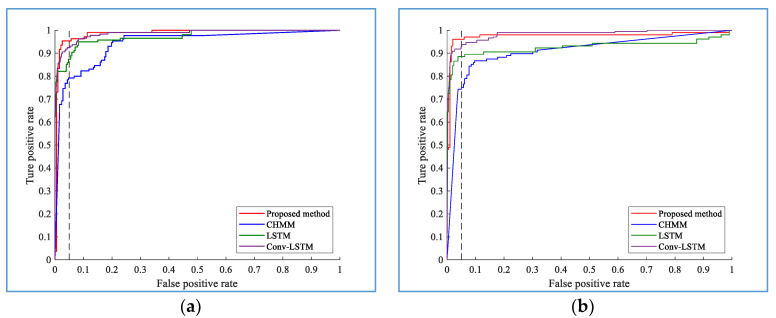
Receiver operating characteristic (ROC) curves for TL (**a**) and TR (**b**) based on the continuous hidden Markov model (CHMM) and the proposed hybrid algorithms at time *t_s_*. The dashed line shows the true positive rate around a false positive rate of 5%.

The performance of the proposed hybrid model for turning intention recognition combined with the online prediction algorithms and deep learning method is compared with the conventional machine learning method CHMM, and the LSTM network with the same architecture, and the LSTM with a similar structure with convolutional layer added (Conv-LSTM). Figure 15a,b shows the recognition results of the TL and TR maneuvers at the time of *t_s_*, respectively. Because the driver has not performed the turning maneuver before *t_s_*, the CHMM does not accurately recognize the maneuver. The ROC curves clearly show the superiority of the proposed hybrid approach over the CHMM-based, LSTM-based, and Conv-LSTM-based algorithms. Specifically, the recognition accuracy of the proposed method at the turning moment *t_s_* is 95.37% for TL with an AUC of 0.9733 and 96.07% for TR with an AUC of 0.9662. In comparison, the recognition accuracy of the CHMM is only 79.23% for TL with an AUC of 0.7968 and 74.33% for TR with an AUC of 0.7651. It can be seen from the figure that the Conv-LSTM network can also achieve better performance, which is better than that of the CHMM-based and LSTM-based algorithms, but is inferior to Bi-LSTM. This result reveals that the superiority of the deep learning method, and also reflects the good time series modeling capabilities of the Bi-directional LSTM in the turning case.

**Figure 16:**
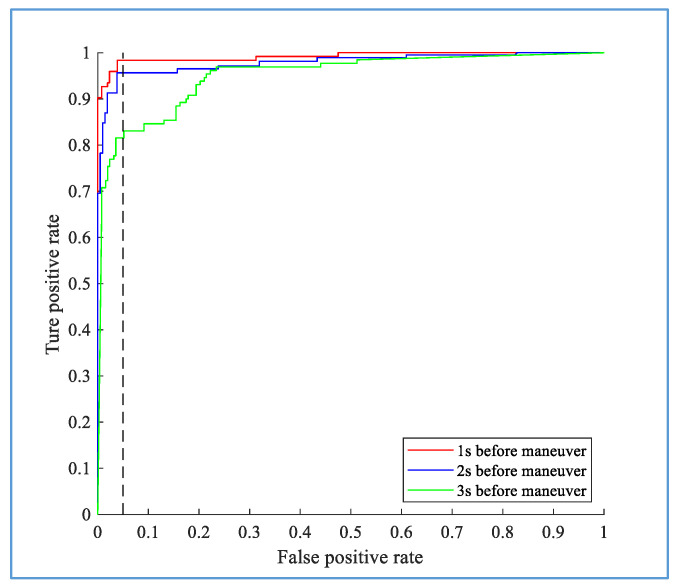
Performance of the proposed method at different times before the actual maneuver for the TL.

**Figure 17:**
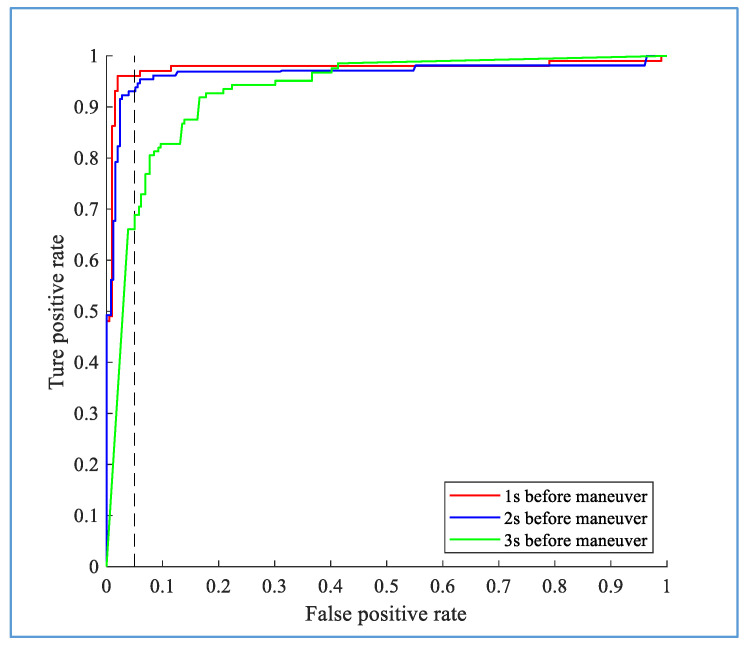
Performance of the proposed method at different times before the actual maneuver for the TR.

The online prediction algorithm uses historical information to predict a future event; the LSTM network has strong capabilities for context modeling, sequential learning, and other nonlinear time series modeling. The Bi-LSTM analyzes data from both ends of the sequence and considers the effect of reverse timing, which improves its predictive ability. Therefore, the proposed hybrid method has strong prediction performance. Figure 16 and Figure 17 show how quickly the proposed method can predict the intention of the driver before the initiation of the TL and TR maneuvers. The results indicate that for the TL and TR behaviors, the model has an average accuracy of 94.2% at 1 s before the maneuver, 93.5% at 2 s before the maneuver, and 74.5% at 3 s before the maneuver.

**Figure 18:**
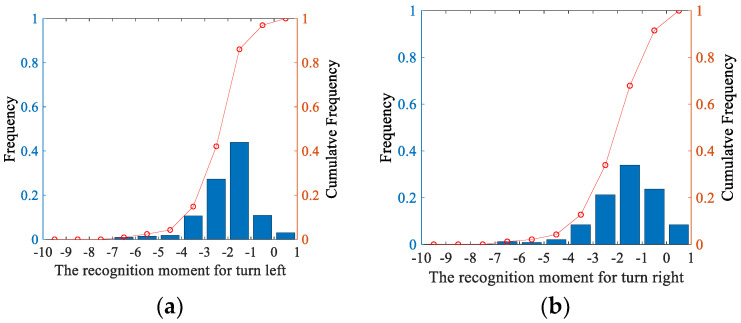

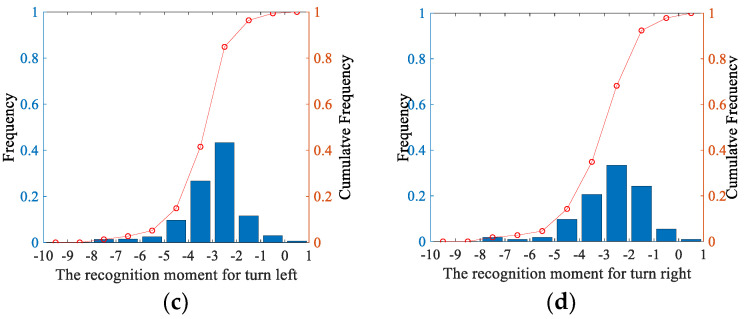
Statistical distribution for the correct recognition result. (**a**,**b**) shows the driving behavior recognition time: (**a**) for TL, (**b**) for TR. (**c**,**d**) are the statistics of the recognition time of the TL and TR maneuvers, respectively, using the proposed hybrid method.

The time to recognize the turning behavior is very critical for ADAS or autonomous vehicles. The earlier the intent of the surrounding vehicles is recognized, the higher the probability is of achieving safe and comfortable driving. Figure 18 shows the driving behavior recognition time; (a) shows the recognition time for TL, (b) shows the recognition time for TR. The data are obtained using only Bi-LSTM to identify the samples. The results shown in (c) and (d) are the statistics of the recognition time of the TL and TR maneuvers, respectively, using the proposed hybrid method. For the TL maneuvers, it is observed in Figure 18a,c that the cumulative frequency of the recognition time of the Bi-LSTM is 86% at 1 s before *t_s_*. The cumulative frequency of the proposed method is 84.8% and 96.36%, respectively at 2 s and 1 s before *t_s_* by using the proposed hybrid method. For the TR maneuvers, we obtain similar results. The use of the hybrid method does not reduce the recognition accuracy much but increases the recognition time. The results show that the proposed hybrid approach provides early recognition of the intention of surrounding vehicles approaching the intersection.

The calculation time of the proposed method for a single sample is a crucial factor in determining whether the method is suitable for real vehicle experiments. Table 3 shows that the average runtime of the hybrid method, whose magnitude is 10 to the negative power of 2 s, indicating that the proposed method meets the real-time requirements of the system.

## 5. Conclusions

In this work, a hybrid approach that combines time series prediction with deep learning networks is proposed to predict the intention of surrounding vehicles when approaching an intersection to improve driving safety. The performance of the proposed hybrid approach is verified using real natural driving data. The driving intention is predicted accurately by the proposed model with an average accuracy of 74.5%, 93.5%, and 94.2% at 3, 2, and 1 s, respectively, before the surrounding vehicles initiate the turning maneuver. The proposed approach can be used to alert drivers of human-driven vehicles of possible safety risks when entering an intersection or to plan a safe and comfortable driving path for ADAS. In the prediction stage, the trajectory, speed, and acceleration of the vehicle are predicted, and the lateral state parameters of the vehicle are derived based on the predicted value, including the lateral speed, lateral acceleration, and heading angle. In the behavior recognition stage, the output of the online prediction algorithm and its derived parameters are input into the Bi-LSTM behavior recognition model to obtain the behavior recognition result.

The use of time series prediction enables the proposed method to perceive the future driving trajectory and driving state of the vehicle. The OGD optimizes in the ARIMA algorithm allows for the online prediction of the vehicle trajectory and state. Due to the RNN, the Bi-LSTM has strong modeling ability, and the hybrid method has excellent predictive ability.

In actual intersections, Lidar or millimeter-wave radar can be used to detect the vehicle’s trajectory, speed, and acceleration; therefore, the proposed hybrid approach may be achievable. In future studies, additional analysis and implementation will be performed to achieve faster detection and higher recognition rates of the intention of the surrounding vehicles after including additional information from the infrastructure, such as traffic light information.

## Figures and Tables

**Table 1:** Summary statistics.

Dataset	Time Period	Going Straight	Left-Turn	Right-Turn	Total
Lankershim	8:30–8:45 a.m.	341	265	315	921
Lankershim	8:45–9:00 a.m.	341	302	339	982
Peachtree	2:45–1:00 p.m.	151	218	173	542
Peachtree	4:00–4:15 p.m.	143	254	151	548
Total	1 h	976	1039	978	2993

**Table 2:** The root mean square error (RMSE) for vehicle’s position, speed, and acceleration in straight through and turn scenarios.

Scenarios	Lateral Position (m)		Longitudinal Position (m)		Speed (m/s)		Acceleration (m/s^2^)	
	RMSE	MAPE (%)	RMSE	MAPE (%)	RMSE	MAPE (%)	RMSE	MAPE (%)
GS	0.0932	1.119	0.1093	1.028	0.1635	1.227	0.2381	0.043
TL	0.2719	0.162	0.1592	0.258	0.3674	0.184	0.1218	0.023
TR	0.1168	0.026	0.3954	0.200	0.1350	0.213	0.4007	0.058

**Table 3:** Average runtime of the recognition.

Model	Online Prediction (s)	Bi-LSTM (s)	Total (s)
Average time	0.0013	0.0150	0.0163
